# The Management of Chronic Pulmonary Aspergillosis: The UK National Aspergillosis Centre Approach

**DOI:** 10.1007/s12281-017-0304-7

**Published:** 2017-11-13

**Authors:** Firas Maghrabi, David W. Denning

**Affiliations:** The National Aspergillosis Centre, Manchester University NHS Foundation Trust, Wythenshawe Hospital, Southmoor Road, Manchester, M23 9LT UK

**Keywords:** Chronic pulmonary aspergillosis, CPA, Long term fungal disease, Drug interactions, Review

## Abstract

**Purpose of Review:**

Chronic pulmonary aspergillosis (CPA) is a serious long-term fungal disease of the lung with a worldwide prevalence. Treatment of CPA is not straightforward given the often-multiple associated co-morbidities, complex clinical picture, drug interactions, toxicities and intolerances.

**Recent Findings:**

First line treatment is oral itraconazole or voriconazole. In the event of intolerance or toxicity, patients may be swapped from itraconazole to voriconazole or vice versa. In the event of resistance or further intolerance, third line treatment with posaconazole could be initiated. In those with pan-azole resistance, short-term courses of intravenous liposomal amphotericin B or micafungin are fourth line therapy, keeping in mind the nephrotoxic effects of amphotericin B.

**Summary:**

The available evidence for current treatments in CPA is limited and based mostly on retrospective cohort studies. There is a real need to raise awareness of this devastating disease to enable early treatment as well as prospective drug trials and studies to identify potential patient factors that correlate with progression, severity and overall outcomes in order to target future therapies.

## Introduction

The *Aspergillus* genus consists of more than 150 species. From epidemiological data, we know that *Aspergillus fumigatus* is the most common species associated with chronic pulmonary aspergillosis (CPA). *A*. *niger*, *A*. *flavus*, *A*. *terreus*, and *A*. *nidulans* have also been implicated in CPA [1]. In contrast to species of *Aspergillus*, *A*. *fumigatus* seems to be the most pathogenic, probably secondary to its ubiquitous nature and resilience in the environment. The mechanistic basis of the pathobiology of *A*. *fumigatus* remains an area of active research and out of the scope of this paper [2]. The small size of *A*. *fumigatus* conidia and fast growth at 37°C enhances its ability to reach the whole airway and germinate [3]. Following interaction and evasion of the host immune responses, the conidia germinate and form a network of hyphae on the interior surface of a cavity, damaging the surrounding parenchyma [4]. In some patients, growth progresses to a full fungal ball or aspergilloma, previously described as mycetoma in the literature [5], a term is more appropriately applied to the subcutaneous infection mycetoma.

**Figure 1:**
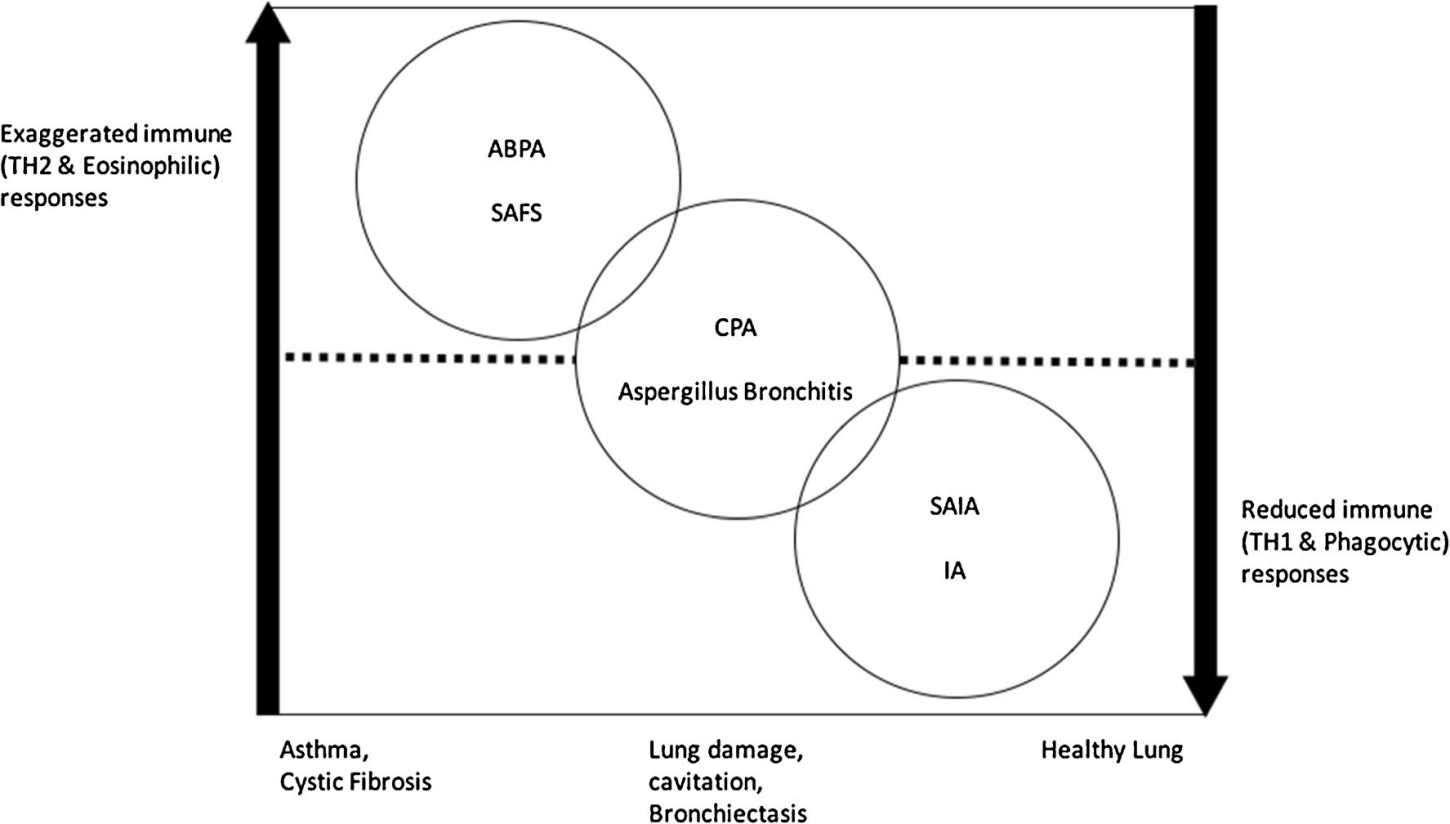
Spectrum of pulmonary aspergillosis and interaction with host immune responses; ABPA, allergic bronchopulmonary aspergillosis; SAFS, severe asthma with fungal sensitisation; SAIA, subacute invasive aspergillosis; IA, invasive aspergillosis

CPA contrasts with invasive aspergillosis (IA) which develops when there is immune dysfunction and allergic bronchopulmonary aspergillosis (ABPA) which occurs in the context of atopy (asthma) with immune hyperactivity. CPA is a serious disease of the lung within the pulmonary aspergillosis spectrum, usually running a progressive course. It affects apparently immunocompetent individuals, usually with a pre-existing lung condition, although that may have been silent: about 5% of cases have no underlying pulmonary or systemic disorder (Fig. 1). Patients with current or prior mycobacterial lung infection (tuberculosis or non-tuberculous mycobacteria) are affected proportionately most often. In addition, a prior diagnosis of ABPA, chronic obstructive pulmonary disease (COPD), sarcoidosis, pneumothorax, lung cancer and previous thoracic surgery are other common antecedents. Diabetes, alcoholism, ankylosing spondylitis and rheumatoid arthritis are also associated, probably due to subtle immune dysfunction [6].

The diagnosis of CPA is often made in patients with suspicious looking radiology, in whom *A*. *fumigatus* is isolated from a respiratory sample by culture. The presence of an aspergilloma is almost certain evidence of CPA. The diagnostic marker detectable *Aspergillus* IgG is strong evidence of infection [7, 8].

The UK National Aspergillosis Centre (NAC) in Manchester receives approximately 130 new patients referred with CPA per year. We have a current cohort of 450 patients under active follow-up [9]. In this paper, the management of CPA from the NAC experience will be explored and discussed.

## Diagnosis

### History

Making the diagnosis of CPA with certainty can be challenging given the similarity the presentation has with other chronic respiratory illnesses (Table 1). Patients can present with a variety of respiratory and constitutional symptoms. The commonest symptoms are shortness of breath, chronic cough, sputum production, chest discomfort, weight loss and fatigue [10–12]. Fatigue can be substantial in CPA and a direct cause for poor quality of life, thought to be driven by uncontrolled chronic inflammation and the overlay of poor physiological baseline from impaired lung function. Patients with CPA also present with significant and life threatening haemoptysis (> 150 ml/day) which is a major cause of morbidity and mortality in CPA [13, 14].

**Table 1:** Diagnostic criteria for different management of chronic pulmonary aspergillosis (CPA). Reproduced from the ERS and ESCMID guidelines for the management of chronic pulmonary aspergillosis [7]

CPA subtype	Diagnostic criteria
Simple aspergilloma [14]	▪ Minimal symptoms or asymptomatic ▪ Single lung cavity containing a fungal ball ▪ Immunological or mycological evidence of *Aspergillus* infection ▪ Immunocompetent patient ▪ No radiological progression over at least 3 months
Chronic cavitary pulmonary aspergillosis, CCPA [7]	▪ Significant symptoms (respiratory and/or constitutional) ▪ One or more lung cavities ± intraluminal material ▪ Immunological or mycological evidence of *Aspergillus* infection ▪ Radiological progression over at least 3 months
Chronic fibrosing pulmonary aspergillosis, CFPA [15]	▪ A complication of CCPA ▪ Severe destruction of two lobes or more ▪ Major loss of lung function ▪ Fibrosis can manifest as consolidation or large cavities with surrounding fibrosis
*Aspergillus* nodule [16]	▪ One or more nodules, which may or may not cavitate. ▪ Main differentials include tuberculosis, lung cancer, and other fungal infections. ▪ Histology is the gold standard for diagnosis ▪ Tissue invasion is not a feature, although necrosis is frequent
Subacute invasive aspergillosis, SAIA [11] Formerly chronic necrotising pulmonary aspergillosis (CNPA)	▪ Mildly immunocompromised patients ▪ Occurs over 1 to 3 months ▪ Radiology could include cavitation, nodules and progressive consolidation with abscess formation ▪ Histology shows lung tissue invaded by hyphae ▪ Microbiological Investigations reflect those in invasive aspergillosis

### Radiology

**Image 1:**
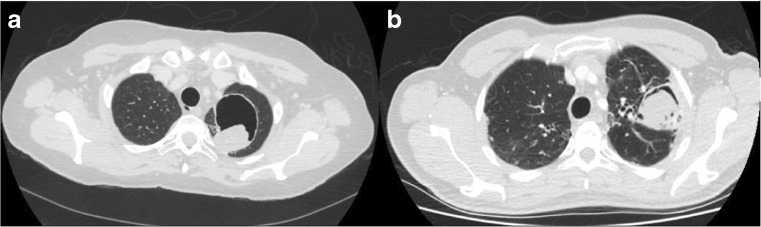
Computed tomography (CT) scan of the thorax shows **a** simple aspergilloma in an asymptomatic patient with a thin-walled cavity and a fungal ball in the left lung. **b** CCPA in a symptomatic patient with cough, shortness of breath and recurrent haemoptysis, and the image shows a left sided thick-walled cavity with pleural thickening and fungal ball

**Image 2:**
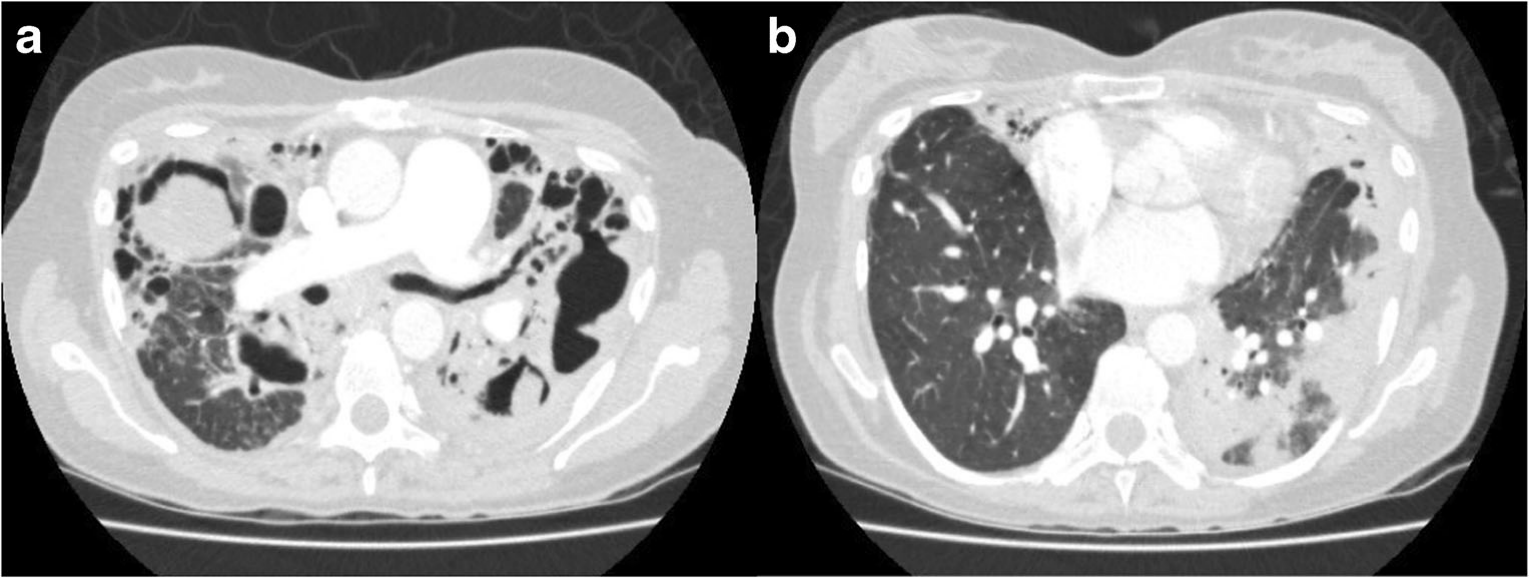
CCPA (bilateral disease) presenting with fatigue, weight loss and cough **a** multiple thick-walled cavities with pleural thickening and intra-cavity material and **b** areas of consolidation more noticeable on the left in the same patient

CPA has many radiological features that can also cause a degree of confusion. CPA can either reveal itself as a simple aspergilloma (image 1a), an *Aspergillus* nodule, or the more common chronic cavitary variant with one or multiple thick or thin-walled cavities, which often contain debris or fungal material and often associated with pleural thickening (images 1b and image 2). A discussion with a specialist thoracic radiologist is often required for full interpretation.

### Chest Imaging

The most important radiological feature of CPA is change over time. Consolidation is often present, could involve an entire lobe and can progress to cavitation and lung volume loss. Typically, CPA affects upper lobes and can appear as single or multiple cavities of various sizes. The cavity wall may be thin (uncommon), irregular or thick. Another common feature in CPA is pleural thickening. A fungal ball is present in approximately half of patients, and could give rise to the classic air crescent sign [17, 18] (which is not specific to CPA) as it could also represent angio-invasive aspergillosis [19].

Computed Tomography (CT) offers better definition than x-ray and enables better characterisation of lung abnormalities. Subtle changes in lung parenchyma can be assessed and monitored over time. Change in radiological appearances can be helpful in monitoring disease activity and progression. In a recent analysis of chest CT scan changes over 6 months of treatment, there was a correlation between response to treatment and reduction of cavity wall thickness, pleural thickness and volume of intra-cavity material. Conversely, cavity size did not correlate well with the improvement in clinical picture [20, 21].

### Microbiology/Mycology

Once CPA is suspected on radiological and clinical grounds, confirming the diagnosis requires microbiological evidence. *Aspergillus* IgG is usually a confirmatory test and aids monitoring of treatment response and relapse of CPA. Commercial tests are 80–96% sensitive and about 85% specific; *Aspergillus* precipitins are less sensitive [22]. Although sensitive, the *Aspergillus* IgG assay is not specific to CPA as it may be positive in those with ABPA, *Aspergillus* bronchitis or sinusitis, or reflect prior infection.

Optimal culture techniques for *Aspergillus* species in sputum have not been well researched, but an increased volume of sputum plated onto fungal media increases yield, and probably testing more than one specimen. *Aspergillus* PCR is a valuable tool used to confirm the presence of *Aspergillus* species in the sputum, its value comes in when culture is negative which could be the case in up to 50% of samples. According to the latest guidance [8], a positive sputum PCR is as good as, if not more sensitive than sputum culture for the diagnosis of CPA. Microscopy and culture of sputum is important to identify *Aspergillus* species, characterise sensitivities and exclude mycobacterial infections. However, not uncommonly obtaining a sputum sample can be difficult. Physiotherapy assistance and induced sputum assist yield [23]. A referral to a bronchoscopist for bronchoalveolar lavage would be the next step. The sample should be tested for fungal PCR and culture. If not ruled out already, a sample should be assessed for acid-fast bacilli and TB culture.

Therefore making a decision based on the overall clinical, radiological and mycological features are important when considering the diagnosis of CPA [24, 22] (Table 2).

**Table 2:** Initial assessment of patients with CPA

Inflammatory markers	
CRP	Raised in CPA, used to monitor progress and response to therapy [25]
ESR/plasma viscosity	
Immunology	
*Aspergillus* IgG	Raised in CPA, used to monitor progress and response to therapy
*Aspergillus* IgE	Raised in ABPA
Microbiology	
Sputum C and S	To rule out resistance, co-infections
Sputum *Aspergillus* PCR	Baseline, probably more sensitive than culture
Imaging	
CXR	Baseline imaging, ideally CT Chest. Repeat after 6 months of treatment, 1–2 yearly thereafter with low dose scanning [2, 18]
CT chest	
Miscellaneous	
St George Quality of Life questionnaire	At baseline, used to monitor response to therapy [26–28]
Weight	
MRC dyspnoea scale	
Pulmonary function tests	
Assessment for immunodeficiency	Poor antibody responses to *S*. *pneumoniae*, *H*. *influenzae* are common [29], as are reduced NK, B and CD4 cell counts [30]

## Treatment

John Hugh Bennett was the first to describe pulmonary aspergillosis in Edinburgh 1842, in a patient who died of CPA [31]. The first case of aspergillosis in the literature to receive intravenous amphotericin B treatment for CPA was in 1957 reported by Kelmenson [32]. However, it was not until 1988, that an oral antifungal agent showed any success in treating CPA, this first successful agent was itraconazole [33].

### First Line

The aim of the treatment depends on the subtype of CPA and clinical situation. First, in patients with stable and asymptomatic simple aspergillomas over a monitoring period of 6 months to 2 years do not require antifungal treatment. However, in symptomatic patients, especially presenting with severe haemoptysis, surgical resection would be the definite cure in this situation.

**Figure 2:**
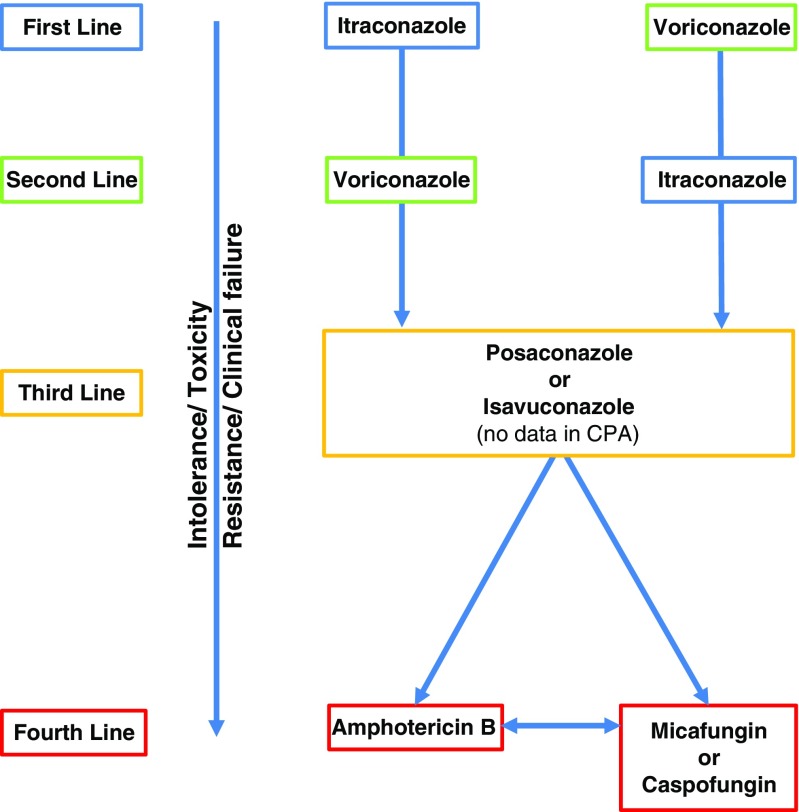
Medical treatment algorithm for CPA

Second, for other types of CPA, oral triazoles are the mainstay of therapy (Fig. 2). The aim could be to cure as in cases of *Aspergillus* nodules [16], or for CCPA and CNPA to improve overall symptoms and so improve quality of life, reduce progressive fibrosis and minimise haemoptysis [26]. This group of drugs are reasonably well tolerated and are active against *Aspergillus* species in vivo and in vitro, with the one exception of fluconazole, which has no activity against *Aspergillus* species and so no role in the treatment of aspergillosis. Their principal mode of action is inhibiting the conversion lanosterol to ergosterol, which in effect disrupts the *Aspergillus* cell membrane.

#### Itraconazole

Over the past 30 years, itraconazole has been adopted widely as first line treatment for CPA given its availability and modest cost. In 2013, Agrawal [34] reported an overall improvement of 77% after 6 months of Itraconazole treatment when compared to standard supportive care, which showed a response of 37%. This rate is close to the response rate in previous literature, which was between 38 and 93% with mean overall response of 63% [35].

However, Itraconazole is not free from side effects and can cause considerable toxicity reportedly in 40–50% of patients. Adverse effects include gastro intestinal upset, hair loss, peripheral neuropathy, hypertension and ankle oedema which may or may not be an early sign of congestive heart failure (Table 3).

**Table 3:**
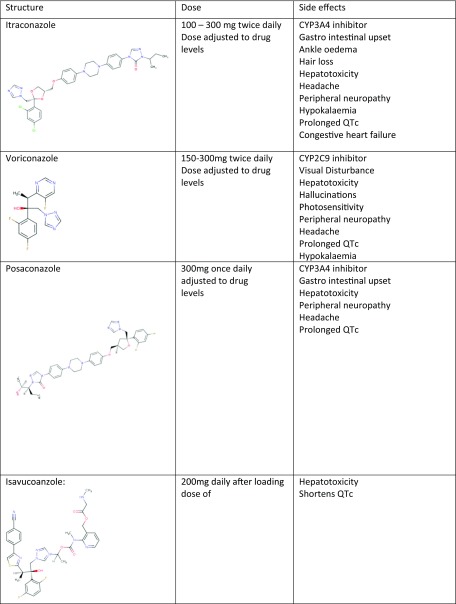
Summary of structure, dosing and side effects of oral triazoles.

Importantly, itraconazole is a potent inhibitor of CYP3A4 and has great potential for drug–drug interactions; therefore, caution and a thorough drug history are required when starting itraconazole. The induction of metabolism by rifampicin and rifabutin is so profound that itraconazole therapy is pointless. Rhabdomyolysis has been reported in patients on the combination of itraconazole and statins [36]. Prolonged QTc interval leading to Torsade de points has also been reported when co-administered with methadone [37] and terfenadine, but not the newer antihistamines [38]. Treatment will often require adjustments to the non-azole treatment and switching to a non-CYP3A4 metabolised alternative. Itraconazole also interacts with inhaled corticosteroids and leads to iatrogenic Cushing syndrome [39] and secondary adrenal insufficiency, especially with fluticasone [40]. Furthermore, protein pump inhibitors and H2 receptor antagonists significantly reduce the absorption of Itraconazole by increasing gastric pH; therefore, further monitoring is required.

At the National Aspergillosis Centre, itraconazole is usually started at a dose of 200 mg twice daily, or in those over 70-years-old 300 mg daily. Levels are routinely monitored during the course of treatment, firstly, after 2–4 weeks on treatment, and then 3–6 monthly according to the initial levels. Various generic brands of itraconazole have different bioavailability so “therapeutic levels” with one brand may go up or down with another, leading to development of resistance and/or clinical failure. Therefore, any unexplained changes in levels require special consideration and enquiry about recent change in brands [41] and we encourage patients to stick on the same brand.

### Second Line

#### Voriconazole

Voriconazole was first reported to treat CPA in the 1990s [11]. Similar to itraconazole, voriconazole is an inhibitor of the CYP2C9 enzyme, thus caution and drug interaction checks are required before commencement. Voriconazole has a reported 6 months overall response rate of 61% [42]. Importantly, adverse reactions are more common with voriconazole, especially visual disturbance, which is usually transient and reversible; yet, papilledema and optic neuritis have been described. Furthermore, hepatotoxicity is a common reason for discontinuation of voriconazole, but usually reversed on discontinuation. Voriconazole is also associated with photosensitivity with a reported increased risk of developing skin malignancies in immunocompromised patients, but rarely in CPA patients [43]. Warning patients about this potential adverse effect is required, especially the regular use of high factor sun block for skin protection should be emphasised [44]. Persistent skin erythema over many months, despite sun protection is a reason to change therapy.

Voriconazole is initiated at 150–200 mg twice daily with close monitoring of levels similar to Itraconazole. Many younger patients require dose escalation to 250 or 300 mg twice daily.

### Third Line

Not uncommonly, patients require switching to third line medication, due to either drug intolerance, drug toxicity, resistance or clinical failure.

#### Posaconazole

Posaconazole is used as third line therapy in CPA. Reported overall success is similar to itraconazole and voriconazole at 61% at 6 months [35]. Adverse effects are far less common than voriconazole. The high cost for long-term use remains the main barrier to the wider use of posaconazole. Drug interactions are fewer than with voriconazole and itraconazole.

#### Isavuconazole

Isavuconazole is the most recently introduced triazole that is FDA approved for the treatment of IA and mucormycosis. Studies have shown non-inferiority to voriconazole for IA, with the added benefit of better tolerability and less adverse effects [45]. However, the jury is still out for Isavuconazole when considering it as a treatment option for CPA, as there are no published data. In patients with prolonged QTc, isavuconazole may be the treatment of choice.

### Fourth Line (Intravenous Treatment)

Intravenous therapy is often used either as last resort following clinical failure, resistance or drug toxicity. Intravenous therapy could also be used as first line treatment in exceptional situations or severe cases at presentation (Table 4).

**Table 4:**
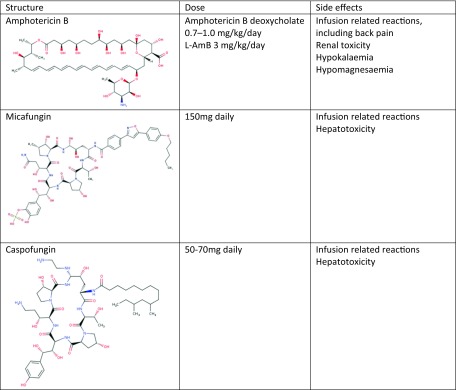
Summary of structure, dosing and side effects of amphotericin B and echinocandins

#### Amphotericin B

Amphotericin B targets ergosterol in the *Aspergillus* cell membrane causing cell death. At the National Aspergillosis Centre, L-AmB is the preferred formulation, given the reported reduced rate of renal toxicity and better tolerance.

A retrospective analysis of outcomes in 71 patients with CPA treated with L-AmB at the National Aspergillosis Centre was recently reported [46]. An induction course was given to newly diagnosed patients with CPA who were very ill, the rationale being avoidance of delays in therapy if the resistance status was unknown and to stimulate the immune response by upregulation of TH1 responses. The median dose given was 3 mg/kg/day for 21 days. Overall response to the first dose of L-Amb was approximately 74%. Twenty patients received two to four courses with a median interval of 6 months between each course. The overall response in this group was approximately 77%.

Intermittent long-term therapy was given to five patients with CPA who had developed pan-azole resistance or who were intolerant of triazoles. The mean dose of L-AmB given was 4 mg/kg/dose given three times a week. A clinical response was evident in all patients; however, four patients eventually failed long-term therapy and two died from respiratory failure. There was a significant fall in eGFR following the first dose of L-AmB, potentiated with further doses of L-AmB affecting 75% of patients.

#### Echinocandins

Echinocandins are large molecules that inhibit *β*-(1, 3)-D-glucan synthase, damaging the fungal cell wall. However, they are fungistatic for *Aspergillus*, affecting on the growing hyphal tips and hence reserved for patients who have failed prior therapy or have renal impairment that would exclude the use of L-AmB as an option [47].

The efficacy of micafungin over 4 weeks is reportedly similar to voriconazole in the treatment of CCPA and SAIA, which could reach 68%, with the added benefit of reduced side effects and drug interactions. Courses are usually given for duration of 3–12 weeks [48]. Caspofungin, another echinocandin has been used in CPA as salvage therapy; however, data for its use beyond small case series (in combination with voriconazole) for this condition is not available [49].

## Prevention of Bacterial Infection

Patients with CPA suffer a high rate of bacterial infection. Many have poor antibody levels against *Streptococcus pneumoniae* and *Haemophilus influenzae* and some respond to immunisation. We administer two doses of Prevanar 13 and one dose of Menitorix to all those with low pneumococcal and *Haemophilus* antibody and check responses [29].

## Bronchial Artery Embolization

Bronchial artery embolization (BAE) is a minimally invasive procedure used to control massive or recurrent haemoptysis as a bridge to more definite therapy. It is also performed for patients who are too unwell to undergo surgery [50]. The immediate success of the procedure is reported to be above 80%. However, repeated interventions are often required, especially if fungal infection is not controlled, as recurrence of haemoptysis is common. Recurrence could be due to recanalization of the embolised vessels, development of collaterals or disease progression [51]. Major complications are rare (< 1%) and include dissection of a bronchial artery, bronchial arterial perforation by a guidewire, transient quadriplegia, transient ischaemic attack, stroke or disseminated infection [52]. Minor complications are more common (30%) and include chest pain, dysphagia and fever [53].

## Surgery

Surgery remains an option in CPA either as first line therapy in *Aspergillus* nodules, and Simple aspergilloma. In two large case series [14, 54] looking at surgical outcomes in patients with CPA who underwent surgery, the commonest indications for surgery were recurrent haemoptysis, cough and increased expectoration. Lobectomy is the commonest procedure performed. Preparation for and risk assessment prior to surgery is important and described in detail by Farid et al. [14].Recurrence following surgical resection, especially in CCPA remains an issue.

## Conclusion

CPA has emerged as a global issue over the past 30 years or so. Carrying 5 year mortality figures similar to some malignancies [28], which make it a devastating, long standing and complex condition. Available treatments are limited in what they could offer, especially in patients with complex disease where palliation is the only realistic option. However, current therapies can improve overall symptomatology and quality of life [26]. Therefore, successful management requires early recognition and a multi-disciplinary approach in order to achieve the best outcome for our patients.
